# Neurophysiological Tools to Investigate Consumer's Gender Differences during the Observation of TV Commercials

**DOI:** 10.1155/2014/912981

**Published:** 2014-07-23

**Authors:** Giovanni Vecchiato, Anton Giulio Maglione, Patrizia Cherubino, Barbara Wasikowska, Agata Wawrzyniak, Anna Latuszynska, Malgorzata Latuszynska, Kesra Nermend, Ilenia Graziani, Maria Rita Leucci, Arianna Trettel, Fabio Babiloni

**Affiliations:** ^1^Department of Physiology and Pharmacology, “Sapienza” University, Piazzale Aldo Moro 5, 00185 Rome, Italy; ^2^BrainSigns s.r.l., Via Sesto Celere 7c, 00152 Rome, Italy; ^3^Department of Anatomy, Histology, Forensic Medicine and Orthopedics, “Sapienza” University, Via Borelli 50, 00185 Rome, Italy; ^4^Department of Economics and Marketing, “IULM” University, Via Carlo Bo 1, 20143 Milan, Italy; ^5^Faculty of Economics and Management, University of Szczecin, Adama Mickiewicza 64, 71-101 Szczecin, Poland

## Abstract

Neuromarketing is a multidisciplinary field of research whose aim is to investigate the consumers' reaction to advertisements from a neuroscientific perspective. In particular, the neuroscience field is thought to be able to reveal information about consumer preferences which are unobtainable through conventional methods, including submitting questionnaires to large samples of consumers or performing psychological personal or group interviews. In this scenario, we performed an experiment in order to investigate cognitive and emotional changes of cerebral activity evaluated by neurophysiologic indices during the observation of TV commercials. In particular, we recorded the electroencephalographic (EEG), galvanic skin response (GSR), and heart rate (HR) in a group of 28 healthy subjects during the observation of a series of TV advertisements that have been grouped by commercial categories. Comparisons of cerebral indices have been performed to highlight gender differences between commercial categories and scenes of interest of two specific commercials. Findings show how EEG methodologies, along with the measurements of autonomic variables, could be used to obtain hidden information to marketers not obtainable otherwise. Most importantly, it was suggested how these tools could help to analyse the perception of TV advertisements and differentiate their production according to the consumer's gender.

## 1. Introduction

In the last two decades, different techniques and devices have been developed to inspect thoughts and feelings by measuring the neuroelectric brain activity through advanced high-resolution EEG methodologies [1–8]. Since last 10 years, unsolved problems and questions related to the evaluation of economic transactions reached the neuroscience labs. Therefore, neuroscience researchers began to cooperate with economists in order to evaluate the brain activity during the generation of economic value judgments. How cognitive neuroscience could integrate the economic sciences on the basis of these last decades of common research? First, cognitive neuroscience deconstructs the picture of perfectly rational humans, deliberating their choices by careful and rationally weighted costs and benefits until a deliberative equilibrium is reached. Although humans are definitely capable of conscious deliberation, many, if not most, economically relevant decision processes are characterized by certain other features such as automatic, fast, and effective cognitive processes, which are not under direct volitional control [9]. Second, as human beings, we are under the influence of unconscious and finely tuned affective mechanisms, which often play a decisive role in action [10–12]. Third, many of these processes have been efficiently shaped by evolution in order to serve social purposes [13–15]. Thus, decision-making and evaluation in economic contexts will be influenced by mechanisms dedicated to social interaction [16]. Hence, the incorporation of neuroimaging into the decision-making sciences has generated the interest not only of economists and academic contexts but also of marketing professionals and companies acting in the market. To now, the most popular brain imaging method adopted in the neuromarketing field is the functional magnetic resonance image (fMRI), a technique which returns a sequence of well refined images of the cerebral activity by measuring the level of oxygen within the cerebral blood flow [17–21]. It is very well known that the hemodynamic measurements, performed by fMRI, are characterized by a high spatial resolution, hence being capable of detecting activations also in deep brain structures such as amygdala or nuclei accumbens. However, the lack of time resolution due to the intrinsic nature of the measured hemodynamic cerebral signals, and the need for experimental subjects to stay in a tube, makes the fMRI unsuitable for this kind of research especially for the problem in to follow subsecond brain dynamics. Anyway, there are other brain imaging techniques that allow to follow up on a millisecond base the brain activity during the exposition to relevant marketing stimuli, such as electroencephalography (EEG). It is characterized by a spatial resolution of square centimeters but to overcome this problem, high-resolution EEG method (hrEEG) has been developed to enhance the poor spatial information content of the EEG activity. In such a way, the brain activity with a spatial resolution of a square centimeter and the unsurpassed time resolution of milliseconds can be detected [22, 23]. In addition, EEG devices are also relatively inexpensive, robust, and even easy wearable by the subject, making such technology be of deep interest for the evaluation of marketing stimuli in “ecologic” paradigms (e.g., TV commercials, point of sales, and supermarkets).

Nowadays, researchers are attempting to investigate the signs of the brain activity correlated with an increase of attention, memory, and emotional engagement during the observation of commercial advertisements [24, 25]. In fact, indirect variables of emotional processing could be gathered by tracking variations of the activity of specific anatomical structures linked to the emotional processing activity in humans, such as the pre- and frontal cortex (PFC and FC, resp. [26]). The PFC is a cerebral region structurally and functionally heterogeneous but its role in the generation of the emotions is well recognized. Specifically, findings suggest that the left PFC is an important brain area in a widespread circuit that mediates appetitive approach, while the right PFC appears to form a major component of a neural circuit that instantiates defensive withdrawal [26, 27]. In addition, the role of the frontal areas in cognitive processes such as memory and attention in complex tasks is very well known [28, 29]. Moreover, by monitoring autonomic activity such as the heart rate (HR) and galvanic skin response (GSR), it is possible to assess the emotional state of the subject [30, 31]. In particular, it was found that greater left-sided activation predicted dispositional tendencies toward approach, whereas greater right-sided asymmetry predicted dispositional tendencies toward avoidance [32]. In contrast, the frontal asymmetry measure did not predict dispositional tendencies toward positive or negative emotions, suggesting an association of frontal asymmetry with approach-avoidance rather than with valence. Thus, the emerging consensus appears to be that frontal EEG asymmetry primarily reflects levels of approach motivation (left hemisphere) versus avoidance motivation (right hemisphere). More recently, resting EEG, self-report measures of behavioral activation and inhibition system (BAS and BIS) strength, dispositional optimism, and a measure of hedonic tone were collected and correlated with alpha asymmetry measures which yielded significant frontal and parietal asymmetry correlation patterns [33].

The way in which such amount of information from neuroscience could be useful to marketing studies is suggested by the well-known fact that at least the 70% of the new products launched worldwide (including cars, shoes, clothes, etc.), tested by traditional techniques with questionnaires or psychological interviews, fail within the first six months. This happens simply because people do not say (or are not able to say) the truth when interviewed, as respect to product experience or TV commercials. Thus, the application of neuroscience-based methodologies really allows the researchers to gain information related to the unconscious and spontaneous reaction of the consumers in front of the product stimuli not available otherwise.

In this scientific framework, the object of our study was to measure and analyze the brain activity and the emotional engagement that occurs during an “ecologic” observation of TV commercials by using EEG and autonomic variables such as HR e GSR. The final goal was to link variations of indicator based on these neurophysiological activities with cognitive and emotional reactions to the presented TV ads. For this purpose, different cerebral indices have been used to summarize the performed measurements at the level of subjects, later used in the statistical analysis. In particular, we performed a gender analysis to investigate and compare cerebral reactions of Men and Women during the observation of specific TV commercials falling in several categories.

In fact, the experimental questions of the present study were the following: are there any particular EEG activities discriminating the levels of cognitive and emotional processing related to the observation of TV advertisements belonging to different commercial categories? Are there any particular scenes of the video clips which were differently perceived by Men and Women? Is it possible to understand which is the best perceived TV commercial between Women and Men? Such questions were answered through the application of the EEG techniques in a case study. Specifically, we recorded EEG, GSR, and HR of a group of Men and Women while watching a series of TV advertisements which have been selected for belonging to several commercial categories. Thus, we compare the percentage of spontaneous recall and appreciation of such commercial categories for Men and Women. The related neurophysiological indices measuring cognitive and emotional processing are computed and compared between the two experimental groups and within particular scenes of interest of different video clips. Finally, the comparison of two TV commercials belonging to the same category will be performed.

## 2. Methods

### 2.1. Experimental Paradigm

The experimental subjects enrolled in this experiment are asked to comfortably have a seat in front of a computer screen by means of which we present a documentary intermingled with several commercial breaks, the target stimuli of the experiment. During the video the electroencephalographic (EEG), electrocardiographic (ECG), and galvanic skin response (GSR) were collected from the subjects. The signals gathered during the observation of the documentary will be used to estimate the personal baseline activity for each one of the EEG, ECG, and GSR variables estimated for the analysis. During the watching of the video, subjects are not aware that an interview would be held within a couple of hours from the end of the data recording. They are simply told to pay attention to what they will watch. The video consisted in a documentary related to geography, with the aim to elicit no particular emotional engagement. After the neurophysiological recordings, an interview is performed. At this stage, the experimenter asks the subjects to recall spontaneously the commercial video clips they memorized. Then, the experimenter verbally listed the sequence of advertisements presented within the documentary asking the subjects to tell which advertisement they remember. Successively, the experimenter showed on a paper several frame sequences of each advertisement inserted in the movie. Analogously, the experimenter also showed several pictures related to an equal number of advertisements that were not inserted in the commercial break (distractors). This was done to provide the subjects with the same number of distractors when compared to the target pictures. Finally, the experimenter asked subjects to give a score ranging between 1 and 10 according to the level of appreciation they perceived during the observation of each remembered ad (1, lowly pleasant; 5, indifferent; 10, highly pleasant). Percentages of spontaneous recall have been standardized through *Z*-scores computed across values of the twelve TV commercials presented. Pleasantness score of each TV commercial has been standardized through *Z*-scores using mean and standard deviation of single subject ratings. These *Z*-scores related to the spontaneous recalls and pleasantness ratings will be used to select two particular TV commercials that will be presented and analyzed in the following sections.

For each subject, a two-minute EEG segment related to the observation of the documentary has been further taken into account as baseline activity. No specific question related to the documentary was asked during the interview.

### 2.2. Subjects and Stimuli

The whole experimental sample is formed by 28 subjects (12 Women). Written informed consent was obtained from each subject after the explanation of the study. The procedure of the experimental task consisted in observing a twenty-minute long documentary in which we inserted two commercial breaks, after five and fifteen minutes from the beginning of the movie, respectively. Each interruption was formed by six commercial video clips of 30′′ length. The TV commercials we use here for case study were unknown to the subjects and they have been showed only once during the experiment. They have been selected in order to define six commercial categories: Perfume, Consumption, Bank, Sport, Telephone, and Clothing. Randomization of the occurrence of the all commercial videos within the documentary was made to remove the sequence factor as possible confounding effect in the following analysis.

**Figure 1:**
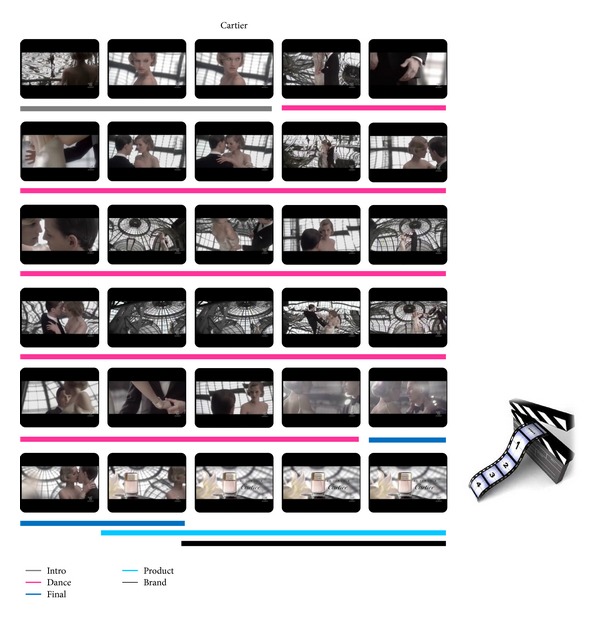
Frame sequence of the Cartier commercial for each second of the video clip. The underlying colors highlight the different scenes in which it is possible to divide the advertisement, as the legend shows. In such segments the average *Z*-scores values for the estimated indices were computed.

**Figure 2:**
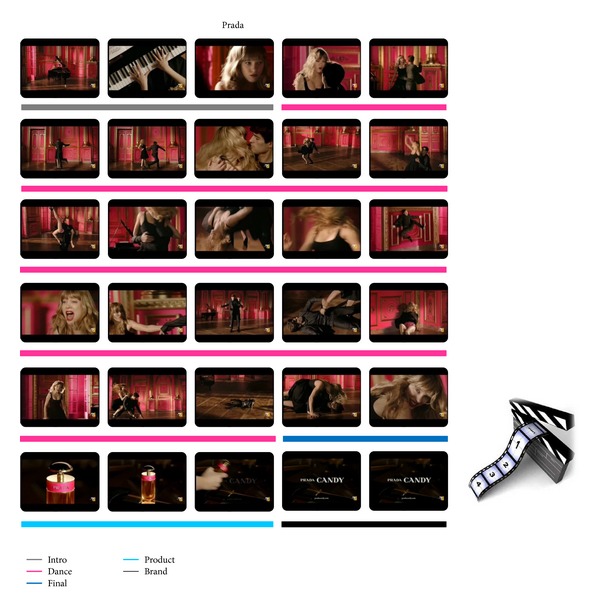
Frame sequence of the Prada commercial for each second of the video clip. The underlying colors highlight the different scenes in which it is possible to divide the advertisement, as the legend shows. In such segments the average *Z*-scores values for the estimated indices were computed.

The two specific advertisements that will be presented and analyzed in the following sections belong to the Perfume category and are aired by Cartier and Prada. The plots of both advertisements have two lovers dancing on a floor as main characters. To inspect the cerebral activity in particular frame segments of the two commercials, they have been properly segmented to define the following scenes: Intro, Dance, Final, Product, and Brand as it is illustrated in Figures 1 and 2.

The TV commercials can be observed at the following links:

 Cartier: http://www.youtube.com/watch?v=D9yVNKEYlDU
 Prada: http://www.youtube.com/watch?v=S_7fBn9enDE.

The cerebral activities have been computed in and compared between the defined frame segments of the two TV commercials.

### 2.3. EEG Recordings and Signal Processing

The cerebral activity was recorded by means of the Digital Brain Electric Activity Mapping KT88-2400 device (Contec Medical Systems Co., Ltd.). Informed consent was obtained from each subject after explanation of the study, which was approved by the local institutional ethics committee. Electrodes were arranged according to the 10–20 International System. Recordings were initially extracerebrally referred to and then converted to an average reference offline. We collected the EEG activity at a sampling rate of 256 Hz and the impedances were kept below 5 kΩ. Each EEG trace was then converted into the EEGlab format in order to perform signal preprocessing such as artefacts detection, filtering, and segmentation. The EEG signals have been band pass filtered at 2–30 Hz and depurated of ocular artefacts by using the independent component analysis (ICA). The EEG data have been rereferenced by computing the common average reference (CAR). Individual alpha frequency (IAF) has been calculated for each subject in order to define four bands of interest according to the method suggested in the literature [29]. Such bands were reported in the following as IAF+*x*, where IAF is the individual alpha frequency, in Hertz, and *x* is an integer displacement in the frequency domain which is employed to define the band ranges. In particular, we focused the present analysis on the following frequency bands: theta (IAF-6, IAF-2), that is, in the frequency band between IAF-6 and IAF-2 Hz, alpha (IAF-2, IAF+2).

These EEG traces were then segmented to obtain the cerebral activity during the observation of the TV commercials and that associated with the documentary (baseline period).

### 2.4. Electrocardiographic Recordings and Signal Processing

We recorded the electrical cardiac activity from the left wrist of all subjects in order to extract the heart rate signal. The recorded signal has been processed in order to compute the peak-to-peak distance from the R-waveform visible on the ECG signal. From this information, we calculated the heart rate tachogram (HR) by computing the reciprocal of the previously defined peak-to-peak distance with in-house MATLAB software. The HR signals of each subject have been averaged to obtain a mean waveform to be compared between experimental conditions.

### 2.5. Galvanic Skin Response Recordings and Signal Processing

The galvanic skin response (GSR) has been recorded with the Neurobit Optima 4 system (Neurobit System, Poland) with a sampling rate of 10 Hz. Skin conductance was recorded by the constant voltage method (0.5 V). Ag-AgCl electrodes were attached to the palmar side of the middle phalanges of the second and third fingers of the subject's nondominant hand by means of a velcro fastener. Before applying the sensors, subjects' skin has been cleaned by the following procedures and suggestions published in the literature [33–35]. GSR has been continuously acquired for the entire duration of the video and then filtered and segmented with in-house MATLAB software. We used a band pass filter with a low cut-off frequency of 0.2 Hz in order to split the phasic component of the electrodermal activity from the tonic one and a high cut-off frequency of 1 Hz to filter out noise and suppress artefacts caused by Ebbecke waves [36].

As explained in the previous section, besides the autonomic activity of the subjects during the observations of the video clips we used a part of the documentary to estimate the mean and standard deviation of the electrodermal activity and the cardiac frequency rate signal in order to compute their *Z*-score variables. These variables have been computed for each TV spot analysed and subject recorded. Specifically, the *Z*-score variables have been computed for the tonic component of the GSR and for the HR signal.

### 2.6. Memorization Index

The EEG signal is filtered in the theta band and, for the subsequent computation, only the left frontal channels have been selected. From the neuroscientific point of view, there is evidence that trace for successful encoding of novel information can be detected by measuring an increase of EEG theta power from the left frontal cerebral regions [28, 37, 38]. Of these channels, we compute the spatial average through the following formulation defining the Memorization Index:

(1) $$\begin{array}{c} \text{MI}=\;\frac{1}{N_{Q}}\underset{i\in Q}{\sum }x_{\theta_{i}}^{2}\left(t\right)={\text{Average}\;\text{Power}}_{\theta_{\text{left},\text{frontal}}}, \end{array}$$

where *x*_*θ*_*i*__ represents the *i*th EEG channel in the theta band that has been recorded from the left frontal lobe. In addition, *Q* is the set of left channels and *N*_*Q*_ represents its cardinality. In such a way, an increase of MI is related to an increase of Memorization. In the following, we will refer to the Memorization Index as Memorization.

### 2.7. Approach-Withdrawal Index

In order to define an Approach-Withdrawal Index (AW) according to the theory related to the earlier introduced EEG frontal asymmetry theory [26], we computed such imbalance as difference between the average EEG power of right and left channels. The formula we used is as follows:

(2) AW=  1NP∑i∈Pxαi2(t)−1NQ∑i∈Qyαi2(t)=Average  Powerαright,frontal−Average  Powerαleft,frontal,

where *x*_*α*_*i*__ and *y*_*α*_*i*__ represent the *i*th EEG channel in the alpha band that have been recorded from the right and left frontal lobes, respectively. In addition, *P* and *Q* are the sets of right channels and left channels and *N*_*P*_ and *N*_*Q*_ represent their cardinality. In such a way, an increase of AW will be related to an increase of interest and vice versa. In such a way, an increase of AW will be related to an increase of interest and vice versa. The AW signal of each subject has been *Z*-score transformed and then averaged to obtain an average waveform. In the following, we will refer to the Approach-Withdrawal Index as Interest.

### 2.8. Emotional Index

The Emotional Index is defined by taking into account the GSR and HR signals. As far as the construction of such variable is concerned, we refer to effects plane [39] where the coordinates of a point in this space are defined by the HR (horizontal axis) and the GSR (vertical axis). Several studies have highlighted that these two autonomic parameters correlate with valence and arousal, respectively (see [40] for a review).

In order to have a monodimensional variable, we describe the emotional state of a subject by defining the following Emotional Index (EI):

(3) $$\begin{array}{c} \text{EI}=1-\frac{\beta }{\pi }, \end{array}$$

where

(4) $$\begin{array}{c} \beta =\{\begin{array}{cc} \frac{3}{2}\pi +\pi -\vartheta & \text{if}\;{\text{GSR}}_{Z}\geq 0,\;{\text{HR}}_{Z}\leq 0,\\ \frac{\pi }{2}-\vartheta \; & \text{otherwise}. \end{array} \end{array}$$

GSR_*Z*_, HR_*Z*_ represent the *Z*-score variables of GSR and HR respectively; *ϑ*, in radians, is measured as arctang(HR_*Z*_, GSR_*Z*_). Therefore, the angle *β* is defined in order to obtain the EI varying between [−1, 1]. According to (2) and (3) and the effect plane [39], negative (HR_*Z*_ < 0) and positive (HR_*Z*_ > 0) values of the EI are related to negative and positive emotions, respectively, spanning the whole effect plane. In the following, we will refer to the Emotional Index as Emotion.

Such cerebral indices take into account that the strong involvement of frontal and prefrontal areas has been already experienced in previous studies performed with high resolution EEG, functional connectivity, and graph theory tools [41–47] as well as in higher cognitive tasks [48–50].

## 3. Results

### 3.1. Behavioral Results

**Figure 3:**
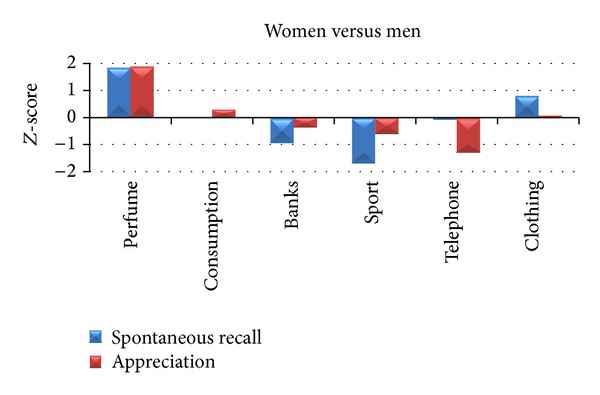
The bar graph shows the difference of average *Z*-score values of percentage of spontaneous recalls (blue) and appreciation (red) between Women and Men in the six commercial categories of analysis. Positive (negative) values indicate higher (lower) spontaneous recall and appreciation for Women (Men).

The recording of the neurometric response included the detection of the EEG signals and HR and GSR parameters on a sample of 28 subjects (22 ± 1.7 years; 12 female). The experimental group has been divided and analyzed by gender. Particularly, we took into account the groups of Women (21 ± 1.7 years) and Men (21.67 ± 1.61 years). The *Z*-score values of percentage of spontaneous recalls and appreciation have been computed for each subject and TV commercial and averaged into the six categories of analysis.  Figure 3 shows the difference of *Z*-score between Women and Men for spontaneous recall and appreciation.

The picture shows the differences related to the percentages of spontaneous recall and appreciation between the groups of Women and Men. Values are represented in *Z*-score. From the bar graph, we can notice that the largest difference between the two genders is related to the observation of TV commercials belonging to the Perfume category, both for spontaneous recalls and appreciation. Particularly, the result is a higher percentage for Women for both spontaneous recalls and appreciation in this category (spontaneous recall: *Z*_women_ = 0.31, *Z*_men_ = −1.54; appreciation: *Z*_women_ = 0.42, *Z*_men_ = −1.49).

In the following, we will analyze the cerebral variables related to the memorization, interest, and emotion for the Perfume category and for the two commercial videos comprising it, Cartier and Prada, by performing a comparison between gender and between video clips, respectively.

### 3.2. Neuroelectrical Results

**Figure 4:**
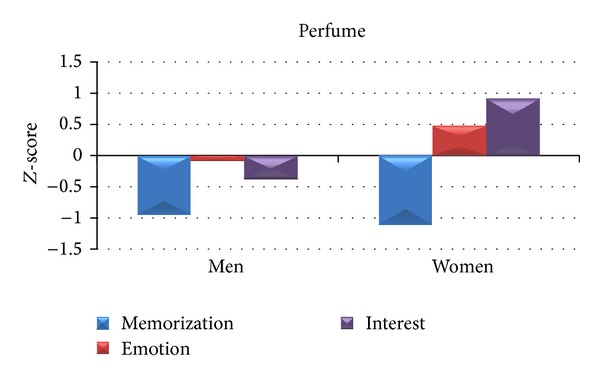
Average *Z*-score values related to the cerebral variables of Memorization (blue), Emotion (red), and Interest (purple) for both groups of Men (left) and Women (right). The average *Z*-scores values refer to the observation of the two TV commercials Cartier and Prada belonging to the Perfume category.

By analysing the cerebral indices of Memorization, Interest, and Emotion for the two commercial advertisements Cartier and Prada belonging to the Perfume category, we observed a different pattern of activation between the two experimental groups, as reported in Figure 4. In particular, the average values of the Memorization between Women and Men do not present high difference, being both negative and close to −1. Men show negative values for both Emotion and Interest, whereas the cerebral activity of Women is characterized by positive values for both Emotion and Interest.

In order to deeper investigate the gender difference during the observation of commercials related to perfumes, we will show a between gender *Z*-score analysis for Cartier and Prada video clips and a between spot *Z*-score comparison for the two genders. All the cerebral variables of Memorization, Emotion, and Interest have been taken into account.

### 3.3. Gender Analysis

**Figure 5:**
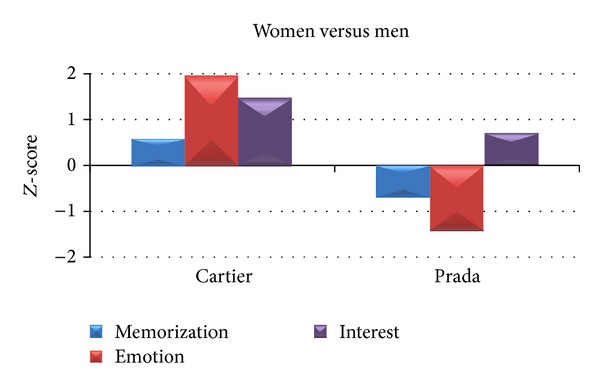
Differences of average *Z*-score values between Women and Men of the cerebral indices of Memorization (blue), Emotion (red), and Interest (purple) for the two TV commercials Cartier (left) and Prada (right). Positive (negative) values indicate higher (lower) values of cerebral indices for Women (Men).

By analysing the variations of the cerebral indices for the two commercials separately, we can observe that the two groups react differently to the observation of the video clips. Specifically, Women present higher values for all the cerebral variables to the Cartier TV spot with respect to Men. Results are illustrated in Figure 5. The highest difference is for the Emotion (*Z*_women_ = 1.91, *Z*_men_ = −0.06). The second TV commercial, Prada, returned higher values of Memorization and Emotion for Men, although the increment of Interest still regards Women.

Since the presented values relate to the observation of the whole video clips, in the following figures we present the values of the cerebral variables within several scenes of interest defined in Section 2. Vertical axes are fixed across figures in order to highlight differences of variations among Memorization, Emotion, and Interest.

**Figure 6:**
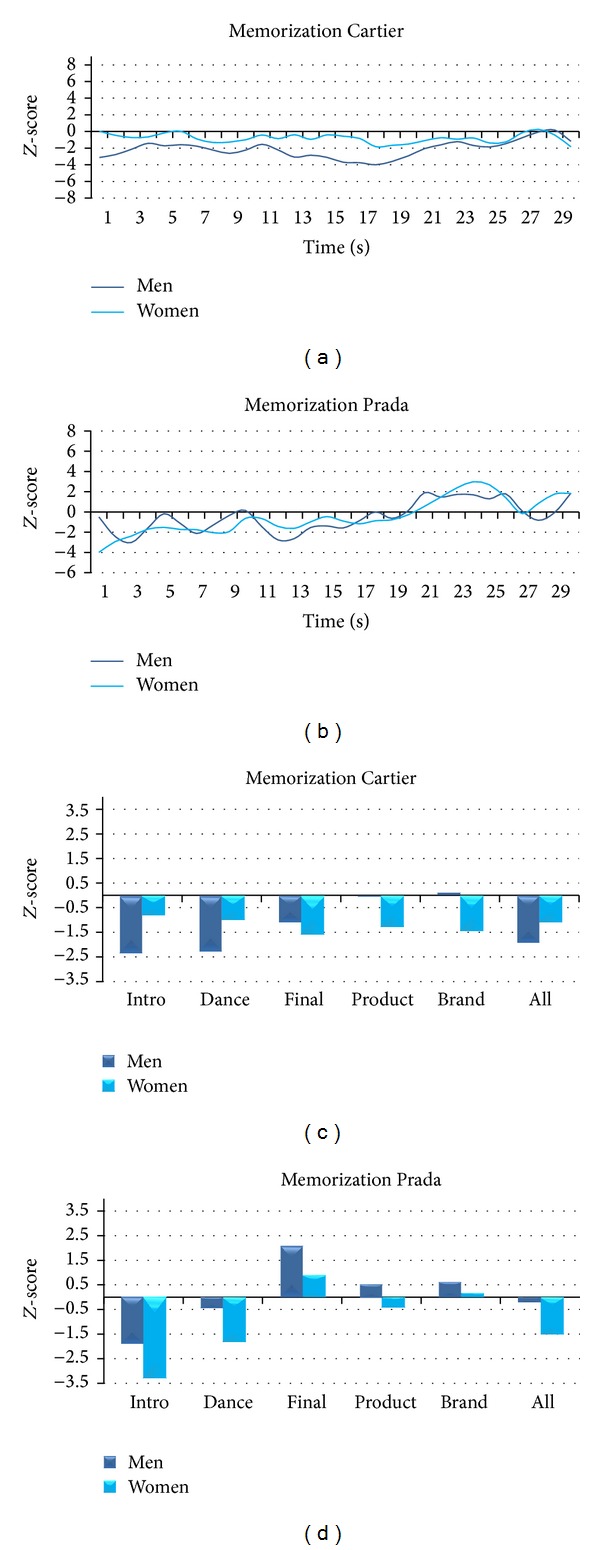
Representation of the variations of the Memorization Index for Cartier and Prada TV commercials for Women and Men. ((a) and (b)) Time course of the average *Z*-score Memorization Index for Women (light blue) and Men (dark blue) for the whole Cartier (a) and Prada (b) TV commercials. ((c) and (d)) Average *Z*-score values of the Memorization Index for the scenes of interest for Women (light blue) and Men (dark blue) for Cartier (c) and Prada (d) TV commercials.

Figure 6 illustrates the average *Z*-score values of Memorization for Women and Men for both Cartier and Prada TV commercials. By analysing the TV spot by Cartier we can observe that Men show a decrease of Memorization for the scenes related to the Intro and Dance (*Z*_intro_men_ = −2.36, *Z*_dance_men_ = −2.28). Conversely, Women present low values of *Z*-scores, in all scene segments of interest, closer to zero. Overall, for both Men and Women, Cartier elicited values of Memorization negative or very close to zero. Instead, Prada returned high values of Memorization for Men in the Final scene (*Z*_final_men_ = 2.11), whereas Women show a decrease of Memorization in the Intro (*Z*_intro_women_ = −3.29).

**Figure 7:**
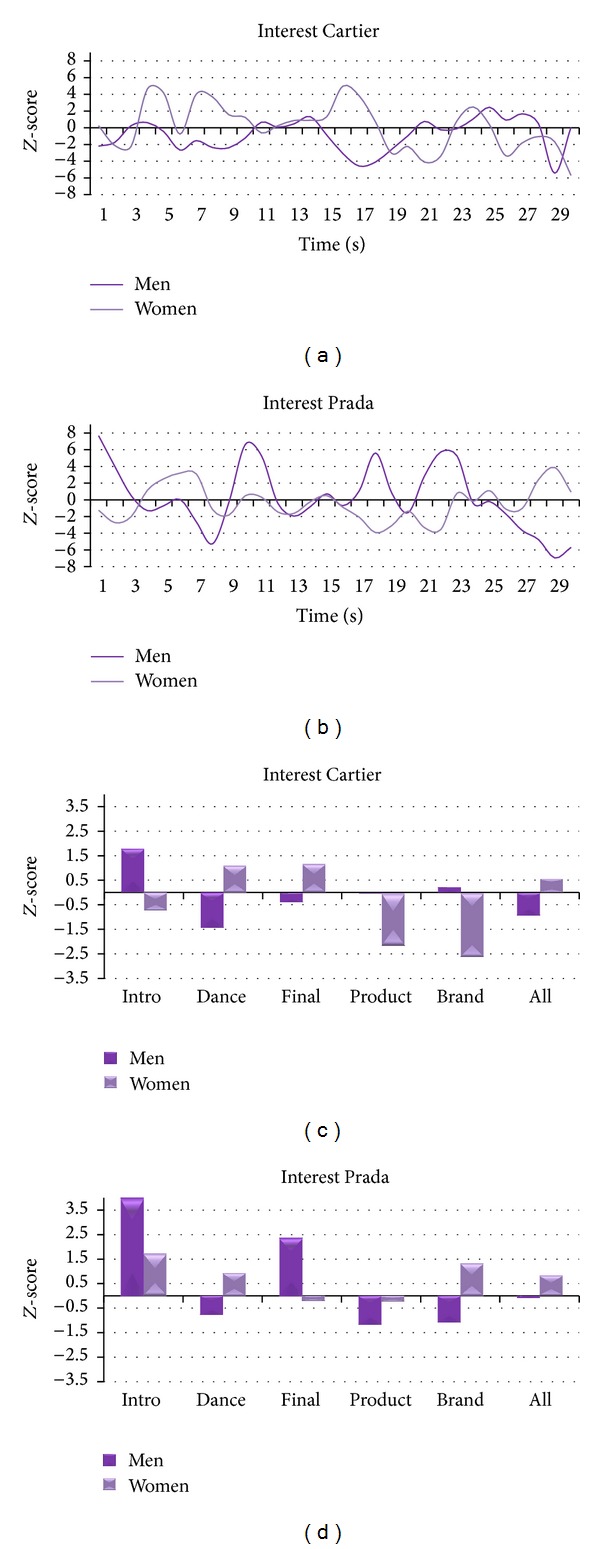
Representation of the variations of the Approach-Withdrawal Index for Cartier and Prada TV commercials for Women and Men. ((a) and (b)) Time course of the average *Z*-score Approach-Withdrawal Index for Women (light purple) and Men (dark purple) for the whole Cartier (a) and Prada (b) TV commercials. ((c) and (d)) Average *Z*-score values of the Approach-Withdrawal Index for the scenes of interest for Women (light purple) and Men (dark purple) for Cartier (c) and Prada (d) TV commercials.

Figure 7 illustrates the average *Z*-score values of Interest for Women and Men for both Cartier and Prada TV commercials. By analysing Cartier, the value of Interest for Women across the whole commercial is positive and higher than Men. However, they present particular scenes in which Interest is negative and lower than Men, such as Product and Brand exposition (*Z*_product_women_ = −2.17, *Z*_brand_women_ = −2.61). By comparing Women and Men segment values within the Cartier clip, we can observe that there are several segments in which Interest is lower for Women with respect to Men, such as Intro, Product, and Brand (*Z*_diff_intro_ = −2.49, *Z*_diff_product_ = −2.19, *Z*_diff_brand_ = −2.84), whereas it is higher during Dance (*Z*_diff_dance_ = 2.52). As to Prada, Men present high values of Interest for Intro and Final (*Z*_intro_men_ = 4.24, *Z*_final_men_ = 2.37), whereas Women returned Interest values close to zero. By comparing the values of the segments between the two genders we find that Interest is higher for Men in Intro and Final (*Z*_diff_intro_ = −2.53, *Z*_diff_final_ = −2.57) whereas it is higher for Women in Brand (*Z*_diff_brand_ = 2.43).

**Figure 8:**
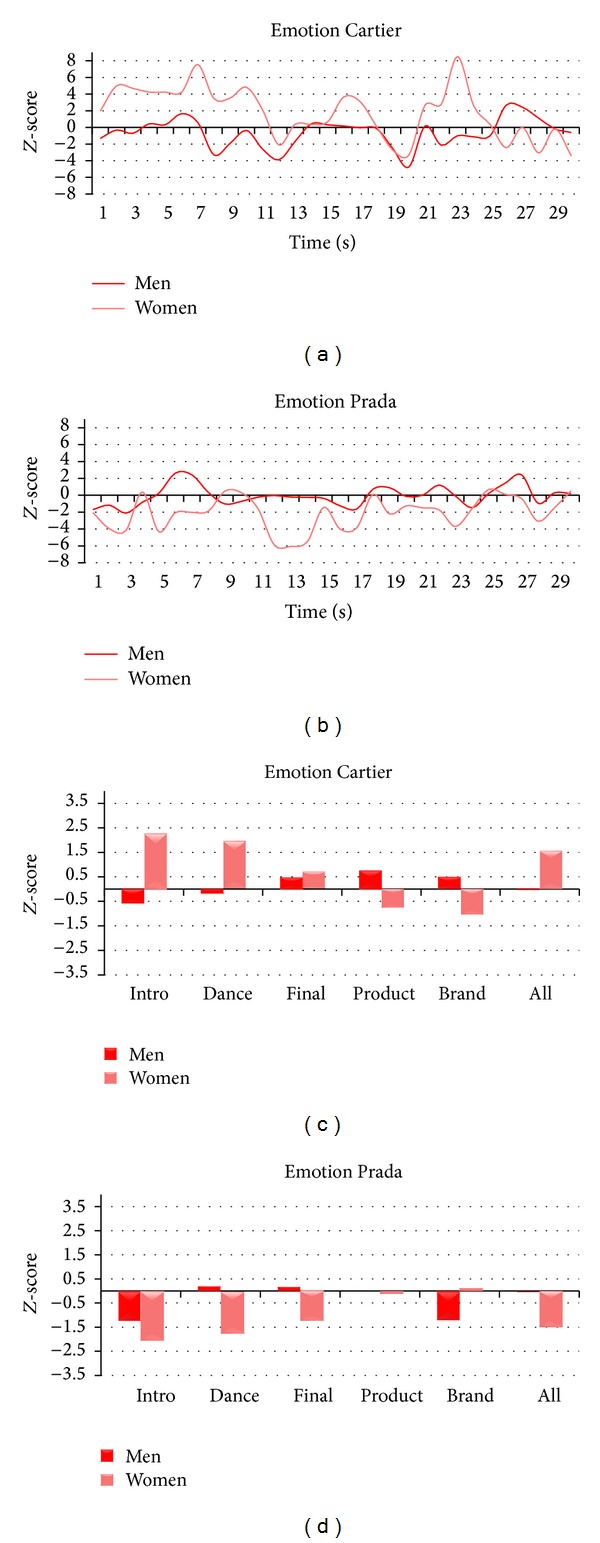
Representation of the variations of the Emotional Index for Cartier and Prada TV commercials for Women and Men. ((a) and (b)) Time course of the average *Z*-score Emotional Index for Women (light red) and Men (dark red) for the whole Cartier (a) and Prada (b) TV commercials. ((c) and (d)) Average *Z*-score values of the Emotional Index for the scenes of interest for Women (light red) and Men (dark red) for Cartier (c) and Prada (d) TV commercials.

Figure 8 illustrates the average *Z*-score values of Emotion for Women and Men for both Cartier and Prada TV commercials. As to Cartier, Emotion value for Women is higher during Intro (*Z*_intro_women_ = 2.29), whereas it does not emerge any increase or decrease of fluctuation of Emotion for Men. In addition, by comparing the values of Emotion between the two groups we find that Women present higher values of Emotion, with respect to Men, for segments of Intro and Dance (*Z*_diff_intro_ = 2.84, *Z*_diff_dance_ = 2.12). As to Prada, all values of Emotion are negative or close to zero both for Women and Men. In particular, Women show a decrease of Emotion during the Intro (*Z*_intro_women_ = −2.04) whereas there is no variation among scenes for Men. However, by comparing Emotion among segments we find that Women present lower values during the Dance (*Z*_diff_dance_ = −1.96).

### 3.4. Comparison of TV Commercials

**Figure 9:**
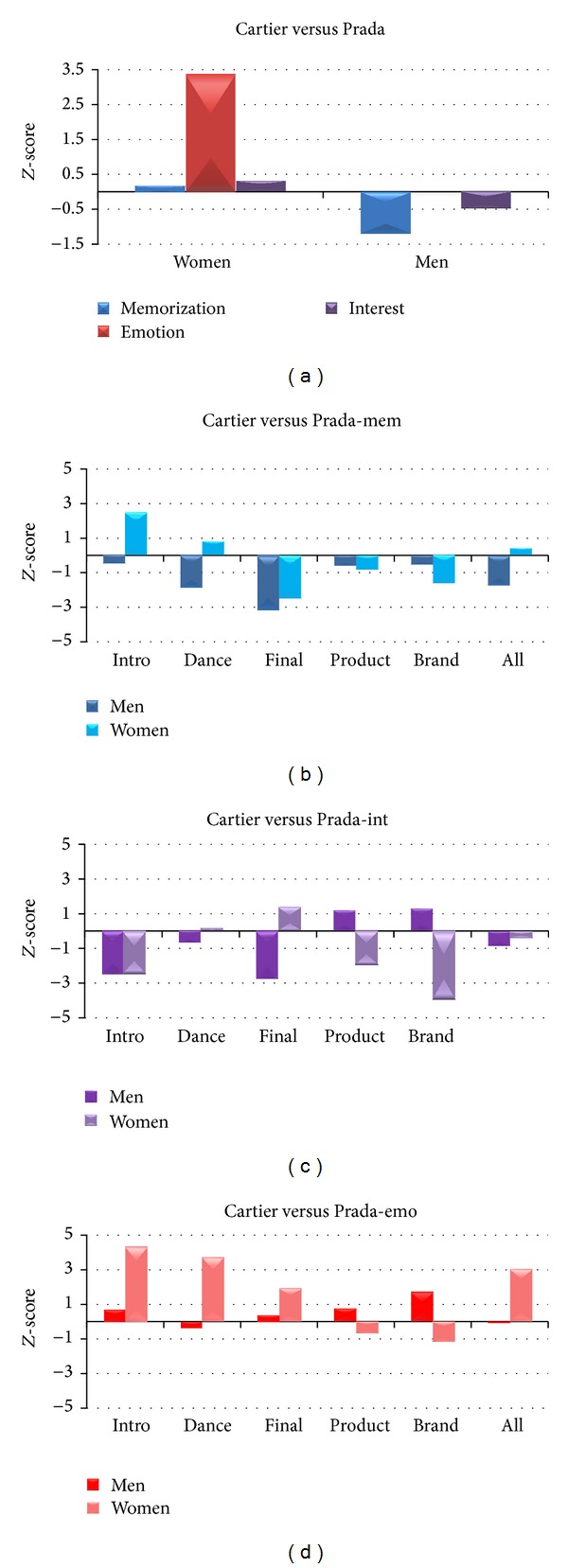
(a) Difference of average *Z*-score values for the cerebral indices of Memorization (blue), Emotion (red), and Interest (purple) between Cartier and Prada TV commercials for both Men (right) and Women (left). ((b), (c), and (d)) Differences of Memorization, Interest, and Emotion across scenes of interest for Women (light colors) and Men (dark colors).

In Figure 9, we are going to present the difference of average *Z*-score values of Memorization, Interest, and Emotion for both Women and Men between Cartier and Prada. In particular, panel A presents differences of cerebral variables between Cartier and Prada for both Women and Men. For this graph bar it is possible to observe that Cartier elicited higher level of Emotion for Women (EmoWomen: *Z*_cartier_ − *Z*_prada_ = 3.39) whereas there is no deviation from zero for Men in all variables. Additional panels highlight difference in the cerebral variables in the specific scenes of the two TV commercials. As to Memorization (Figure 9(b)), Prada elicited high values for both Women and Men in the Final scene (*Z*_final_women_ = −2.48, *Z*_final_men_ = −3.18), while the Intro returned high value for Cartier only for Women (*Z*_intro_women_ = 2.491). As to Interest (Figure 9(c)), we can observe that the Intro by Prada is characterized by higher values both for Women and Men (*Z*_intro_men_ = −2.47, *Z*_intro_women_ = −2.44), whereas the same commercial returns higher value for Women during Product and Brand (*Z*_product_women_ = −1.97, *Z*_brand_women_ = −3.97) and the Final scene for Men (*Z*_final_men_ = −2.75). There is no increment of Interest for Cartier with respect to Prada. As to Emotion (Figure 9(d)), Cartier elicited higher values for Women during the Intro, Dance, and across the whole video clip (*Z*_intro_women_ = 4.33, *Z*_dance_women_ = 3.72, *Z*_all_women_ = 3.05). No deviation from zero for Men between the two commercials.

## 4. Discussion

Behavioral data in terms of spontaneous recall and appreciation returned that the highest difference between Women and Men is related to the observation of two TV advertisements belonging to the category of Perfume. In particular, Women exhibit higher percentage of spontaneous recall and level of appreciation with respect to Men.

The level of Memorization of the two TV commercials analyzed is low for both Women and Men and there is no variation between the two groups. The values of Memorization for Cartier and Prada result lower that the ones of the other commercial categories. As to Emotion, we observed that Women are more engaged during some introductive scenes of the spot by Cartier but less for Prada. In particular, the Dance scene of Cartier gives more emotion for Women while the Dance scene of Prada gives more emotion to Men. The cerebral variable related to the Interest presents a variegated pattern because there are different catching scenes both for Women and Men depending on the TV commercial. By comparing the cerebral variables between the two commercials, we observed that there are no differences of emotion between the two commercials for Men, whereas Women were more engaged during the observation of Cartier video clip. Conversely, the advertisement by Prada returned higher values of Memorization and Interest for both Women and Men.

Overall, the biometric EEG, HR, and GSR recordings returned neurometric indexes linked to the variation of the Memorization, Interest, and emotional involvement of the two experimental groups. Variation of such indices along with the adopted time segments returned information about the ads perception, scene by scene, on the total sample of recorded subjects and on subsamples of such group. This information may be analyzed by providing interesting indications about the efficacy of the different frame sequences or observing original insights related to cognitive and emotional variables. Also, these tools could provide a rational schema useful to guide a reduction of the ads in time, which is often implemented in advertising campaigns after the first detailed creative production, by pointing out the most (less) performing scenes which could be preserved (cut) by a possible time-frame reduction. Such time reduction could be specifically performed and differently adapted to Men and Women [51].

Nowadays, marketers are excited about the use of brain imaging for marketing purposes. First, they hope that neuroimaging may help to refine the possibilities of marketing research improving an efficient trade-off between costs and benefits. This hope is based on the assumptions that people cannot fully articulate their preferences when asked to express them explicitly and that consumers' brains contain hidden information about their true preferences. Such hidden information could, respecting the more recent neuroscience theory, be used to better understand their buying behaviour and meet their needs. Thus, the cost of performing neuroimaging studies would be outweighed by the benefit of improved product design and increased sales. In theory, at least, brain imaging could illuminate not only what people like, but also what they will buy. Thus far, this presented approach to neuromarketing has focused on this postdesign application, in particular on measuring the effectiveness of advertising campaigns. Particularly, we showed that there are results suggesting that it is possible to differentiate the communication according to the gender of the analyzed population. Properly designing and broadcasting two different versions of marketing communication will help to enhance the efficacy and the quality of the message. Moreover, an objective method for reducing the time length of the TV commercial will help the video makers to have a rational basis for cutting ineffective scenes and, hopefully, to create new more appealing TV commercials. Thus, while the emotional and cerebral “engaging” of the video clip is preserved, the cost of airing the video clip could be reduced saving some money by getting the same efficacy (or even more) than in the 30′′ version.

Moreover, neuromarketing research can be implemented even before a product exists, because the assumption that neuroimaging data would give a more accurate indication of the underlying preferences than standard market research studies may be really useful avoiding expensive mistakes. If this is indeed the case, product concepts could be tested rapidly, and those that are not promising were eliminated early in the process. This would allow more efficient allocation of resources to develop only promising products.
